# Epigenetic regulation of macrophage polarization in wound healing

**DOI:** 10.1093/burnst/tkac057

**Published:** 2023-01-17

**Authors:** Cheng Chen, Tengfei Liu, Yuanyang Tang, Gaoxing Luo, Guangping Liang, Weifeng He

**Affiliations:** State Key Laboratory of Trauma, Burn and Combined Injury, Institute of Burn Research, Southwest Hospital, Third Military Medical University (Army Medical University), Chongqing 400038, China; Chongqing Key Laboratory for Disease Proteomics, Chongqing 400038, China; State Key Laboratory of Trauma, Burn and Combined Injury, Institute of Burn Research, Southwest Hospital, Third Military Medical University (Army Medical University), Chongqing 400038, China; No. 906 Hospital of Joint Logistic Support Force of PLA, Ningbo, Zhejiang, China; State Key Laboratory of Trauma, Burn and Combined Injury, Institute of Burn Research, Southwest Hospital, Third Military Medical University (Army Medical University), Chongqing 400038, China; Academy of Biological Engineering, Chongqing University, Chongqing, China; State Key Laboratory of Trauma, Burn and Combined Injury, Institute of Burn Research, Southwest Hospital, Third Military Medical University (Army Medical University), Chongqing 400038, China; Chongqing Key Laboratory for Disease Proteomics, Chongqing 400038, China; State Key Laboratory of Trauma, Burn and Combined Injury, Institute of Burn Research, Southwest Hospital, Third Military Medical University (Army Medical University), Chongqing 400038, China; Chongqing Key Laboratory for Disease Proteomics, Chongqing 400038, China; State Key Laboratory of Trauma, Burn and Combined Injury, Institute of Burn Research, Southwest Hospital, Third Military Medical University (Army Medical University), Chongqing 400038, China; Chongqing Key Laboratory for Disease Proteomics, Chongqing 400038, China

**Keywords:** Epigenetics, Macrophage polarization, Wound healing, Immune microenvironment, Signaling pathways

## Abstract

The immune microenvironment plays a critical role in regulating skin wound healing. Macrophages, the main component of infiltrating inflammatory cells, play a pivotal role in shaping the immune microenvironment in the process of skin wound healing. Macrophages comprise the classic proinflammatory M1 subtype and anti-inflammatory M2 population. In the early inflammatory phase of skin wound closure, M1-like macrophages initiate and amplify the local inflammatory response to disinfect the injured tissue. In the late tissue-repairing phase, M2 macrophages are predominant in wound tissue and limit local inflammation to promote tissue repair. The biological function of macrophages is tightly linked with epigenomic organization. Transcription factors are essential for macrophage polarization. Epigenetic modification of transcription factors determines the heterogeneity of macrophages. In contrast, transcription factors also regulate the expression of epigenetic enzymes. Both transcription factors and epigenetic enzymes form a complex network that regulates the plasticity of macrophages. Here, we describe the latest knowledge concerning the potential epigenetic mechanisms that precisely regulate the biological function of macrophages and their effects on skin wound healing.

Highlights

- Emphasizing insufficient inflammation and defective macrophages in earlydiabetic wound.
- Summarizing the networks of epigenetics and transcription factors.
- Pharmacologicalmodulators targeting epigenetic enzymes to influence macrophage phenotype.

## Background

Macrophages are fundamental innate immune cells in the skin that not only maintain tissue homeostasis but also play an important role in disease. Skin macrophages are derived from two sources, skin-resident macrophages and bone marrow-derived macrophages. In normal skin, only skin-resident macrophages sustain homeostasis and are present at a low density of ~1–2 per mm^2^. In injured skin, bone marrow-derived macrophages play a major role in wound repair.

Three dynamic and overlapping phases make up the classic wound healing process: the inflammation, proliferation and remodeling phases. Macrophages participate in all phases and regulate the wound microenvironment. After injury, monocytes are recruited within 48–96 h, and the number of macrophages peaks at day 3 [1]. The arriving macrophages clear the corpses of neutrophils to avoid a persistent inflammatory state and secrete proinflammatory cytokines to recruit other inflammatory cells [2]. However, the wound microenvironment also influences the heterogeneity of macrophages [3].

Epigenetics refers to environmental factors that influence the transmission of the genome without changing the DNA sequence. The biggest difference between epigenetics and classical genetics is invariable DNA sequences accompanied by persistent and heritable changes in gene expression [4]. The most common epigenetic modifications include DNA methylation, histone posttranslational modifications and noncoding regulatory RNA editing [5]. In programming myeloid development and macrophage phenotype transition, epigenetic modifications form phenotypic discrepancies by selectively inducing expression or repression of a subset of genes. Therefore, understanding the development and function of wound macrophages and their regulatory mode is useful. Recently, several excellent reviews have described epigenetic regulation in wound healing, focusing on the proliferation, migration and differentiation of epidermal stem cells and fibroblasts [6,7]. However, as described above, macrophages are also essential for wound healing.

Herein, we summarize the epigenetic modifications in the monocyte development process that contribute to macrophage heterogeneity. This review emphasizes macrophage heterogeneity regulated by epigenetic modifications in wound healing and may contribute to the promotion of epigenetic modifications as an innovative diagnostic and therapeutic target for wound healing.

## Review

### The role of macrophage polarization in wound healing

**Figure 1:**
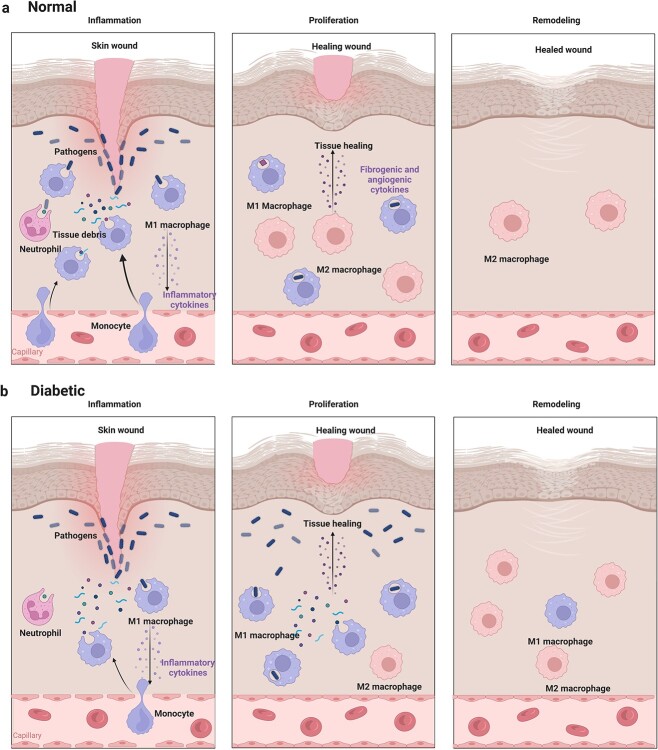
Macrophages in normal and diabetic wounds. (**a**) After injury, the recruitment of bone marrow-derived macrophages (BMDMs) plays an essential role in the inflammatory phase of wound healing. M1 macrophages clear pathogens and damaged tissues to avoid persistent stimulation by damage-associated molecular patterns and pathogen-associated molecular patterns and promote the transformation from inflammation to proliferation. At the remodeling stage, macrophages fade away [16]. (**b**) In diabetic wounds, delayed recruitment and short-lived BMDMs are caused by hyperglycemia, along with an attenuated ability to clear pathogens and damaged tissues [13]. The transformation from inflammation to proliferation and the disappearance of macrophages cannot occur in a timely manner

The immune microenvironment is involved in regulating skin wound healing [8]. At an early stage of wound healing, a proinflammatory reaction is conducive to anti-infection activity in injured tissue and a late pro-healing response is beneficial to wound repair [9]. As the main component of infiltrating inflammatory cells, macrophages show features of remarkable plasticity and longevity, which are integral for shaping the immune microenvironment in the skin wound healing process. Mature macrophages are polarized and classified as a proinflammatory M1 subtype or an anti-inflammatory M2 subtype under the influence of proinflammatory and anti-inflammatory cytokines, respectively, which is called macrophage polarization [10]. M1 macrophages are dominant in the early stage of wound closure and show increased phagocytic activity and secretion of proinflammatory cytokines, such as interleukin (IL)-1, IL-6, IL-12, tumor necrosis factor alpha (TNF-α) and oxidative metabolites, to remove pathogens and damaged tissues [11]. At ~5 days after injury, Th2 cytokines, such as IL-4, IL-13 and IL-10, are responsible for macrophage polarization into the M2-like phenotype, which means transmission from the inflammation to the proliferation stage. M2 macrophages replace M1 macrophages and become the protagonist in the late tissue-repairing phase and show a polar opposite phenotype by producing anti-inflammatory fibrogenic and angiogenic mediators to limit local inflammation and promote tissue repair [12]. Therefore, most studies focus on the phenotypic switch of macrophages in chronic low-grade inflammation, such as diabetic wounds. Indeed, the timely transformation of macrophages from M1 to M2 determines the speed and quality of wound healing. However, the underlying mechanism leading from acute to chronic diabetic wounds should be given more attention. Macrophages in diabetic mice show a reduced immune response and decreased number in the early stage of wounds, indicating that the inflammation in diabetic wounds cannot effectively clear pathogens and damaged tissues. The immune microenvironment affected by pathogens and damaged tissues attracts more macrophages and persistently stimulates these functionally defective macrophages. After thoroughly clearing the pathogens and damaged tissues, these M1 macrophages can transform into M2 macrophages. The feature of ‘slowly coming into and slowly going out’ in diabetic wound macrophages and the presence of insufficient M2 macrophages in the early stage but excessive M2 macrophages in the later proliferative phase may support this idea [13]. Therefore, enhancing the ability of macrophages to clear pathogens and damaged tissues at the early wound stage in diabetes may be a clinical therapeutic target (Figure 1).

Although traditional M1 and M2 macrophages have been identified, many novel phenotypes cannot be explained by only these two canonical groups. M2-like macrophages can be divided into four subsets according to their function. M2a macrophages are referred to as alternative M2 macrophages, which promote vessel and scar formation. Unlike M2a macrophages, M2c macrophages, also called Mreg-like macrophages, phagocytize excessive matrix to avoid skin fibrosis at the remodeling stage [14]. M2b macrophages are responsible for anti-inflammation activity. Compared with IL-4-activated M2a macrophages, Lipopolysaccharide (LPS)- and adenosine A-induced M2d macrophages express higher levels of IL-10 and Vascular endothelial growth factor (VEGF) and lower levels of TNF-α and IL-12. Moreover, the M2d macrophage subtype represents a model switch from M1 macrophages (LPS) to M2 macrophages (adenosine A) and is an essential subset for angiogenesis [15,16]. Actually, the extracellular milieu of wounds is the determinant of macrophage phenotype and influences transcription factors (TFs) to form a regulatory network that induces macrophage polarization.

### TFs in macrophage polarization

Macrophages are characterized by high plasticity and their heterogeneous members constitute a continuum. M1 (classical) or M2 (alternative) are the extremes of the continuum [17]. To acquire distinct functional phenotypes, the environment stimulates intrinsic terminal differentiation pathways in macrophages. LPS, Interferon (IFN)-γ, IFN-β and GM-CSF induce classical M1 activation, while IL-4/IL-13 or IL-10 skew macrophages toward M2 activation. Transcription factors downstream of these stimuli are essential for the induction of functional cytokines.

NF-κB, which is essential for M1 polarization, is activated by LPS and controls the expression of inflammatory cytokines [18]. NF-κB complexes are formed by two subunits of the Rel family, which includes RelA (p65), RelB, c-Rel, NF-κB1 (p50) and NF-κB2 (p52) [19]. The p65 and p50 heterodimer is the commonly accepted proinflammatory NF-κB, and the homodimers P50-P50 and P52-P52 are inactive and always inhibit M1 polarization. Another TF, an inhibitor of NF-κB (IκB, mainly inhibitor kappa B (IκBα)), also restricts the nuclear activity of NF-κB. IκB also abolishes the transcriptional activity of IFN regulatory factor 1 (IRF1) by altering its interaction with the RelA subunit of NF-kB [20]. Among the nine types of IRF proteins, IRF1 and IRF2 cooperate with NF-kB to promote M1 polarization and block the expression of IL-4 at the same time. However, unlike IRF1, the role of IRF2 in macrophage polarization depends on stimulating factors. IRF2 exhibits inflammatory properties during infection and anti-inflammatory properties during sterile inflammation [21,22]. Along with IRF1, IRF5 and IRF8 also promote M1 macrophage polarization. IRF5 is a critical player in the formation of both IFN-γ- and LPS-stimulated M1-like phenotypes and mediates key inflammatory cytokines such as TNF-α, IL-6 and IL-12 [23]. As a positive TF in M2 macrophage polarization, IRF3 suppresses proinflammatory genes and enhances anti-inflammatory genes by activating phosphatidylinositol-3-kinase (PI3K/Akt) signaling [24], and IRF4 enhances anti-inflammatory genes by competing with IRF5 for binding to Myeloid differentiation factor88 (MyD88) and upregulating signal transducer and activator of transcription 6 (STAT6).

The STAT family consists of seven members (STAT1, STAT2, STAT3, STAT4, STAT5A, STAT5B and STAT6) in mammals [25]. STAT1 is mainly induced by IFN-γ, while STAT3 and STAT6 are induced by IL-10 and IL-4/IL-13, respectively. STAT1 is essential for binding the promoter of IFN-β, CXCL9 and CXCL10 [26]. Under LPS stimulation, IRF3 induces the autocrine signaling of IFN-β to indirectly regulate STAT1 and STAT2 [27]. Then, both recruit IRF9 to activate the inducible nitric oxide synthase (iNOS), major histocompatibility complexII (MHCII) and IL-12 genes. GM-CSF induced phenotype transition is mediated by STAT3 and STAT5. STAT3 shows activity opposite that of STAT1 [17]. As a classic induction factor of STAT1, IFN-γ suppresses the induction of IL-10 and downstream STAT3 activation. STAT6 is activated by IL-4/13, which skews M2 polarization by promoting mannose receptor (Mrc1), peroxisome proliferator-activated receptor γ (PPAR γ) and PPAR δ. STAT4 can be induced by IL-12, LPS and type I IFN and STAT5 can be induced by IL-2. Deletion of STAT4 and STAT5 leads to an increase in M2 macrophages, indicating that these STATs contribute to M1 polarization [28]. Collectively, STAT1, STAT2, STAT4 and STAT5 coordinate M1 polarization, while STAT3 and STAT6 coordinate M2 polarization. Suppressors of cytokine signaling (SOCS) proteins are endogenous inhibitors of STATs and include SOCS1–7 [29,30]. Unlike other TFs, the role of SOCS in macrophage polarization seems more complex and controversial. IL-4-induced STAT6 can upregulate SOCS1 but inhibits SOCS3. On the one hand, SOCS1 enhances PI3K activity, which drives M2 activation. On the other hand, SOCS1 mediates the proinflammatory response (IL-6, IL-12, MHC class II, NO) after LPS stimulation [31]. STAT3 can be activated by proinflammatory IL-6 and anti-inflammatory IL-10. How do these two cytokines show such opposing functions with common downstream TFs? Yasukawa *et al*. reported that SOCS3 specifically inhibits the activation of STAT3 by IL-6 but not IL-10 [32]. However, how the microenvironment regulates the expression of TFs merits further investigation. Recently, the role of epigenetics in the modification of promoter and enhancer regions in TFs has been brought into focus.

### Epigenetic mechanisms that influence the plasticity of macrophages

Epigenetic regulation is the study of heritable phenomena without changes in the nucleotide sequence and plays an essential role in immune cell activation. In macrophages, epigenetic regulations, such as post-translational modification of histones, DNA methylation and noncoding RNA (ncRNA) editing, all participate in the expression of functional molecules (Table 1).

**Table 1:** Function and regulation of epigenetic enzymes

**Enzyme category**	**Family member**	**Function**	**Effect on macrophages**	**Pharmacologic inhibitors to promote the M2 phenotype**
HMEs				
HAT	P300/CBP	Inhibits the NF-κB signaling pathway	Promotes M2 polarization	HATi II, roscovitine, curcumin;
HDAC	HDAC3	Activates IL-6, NO, IFNβ, NOS2;	Promotes M1 polarization	SAHA VPA, butyrate;
	HDAC9	Inhibits PPAR γ expression	Promotes M1 polarization	
	HDAC4	Inhibits the NF-κB pathway	Promotes M2 polarization	
	SIRT1	Inhibits the NF-κB pathway	Promotes M2 polarization	
HMTs	SET7/9	Induces the production of TNF and MCP-1	Promotes M1 polarization	DZNep, MI-2-2, MTA;
	SMYD2	Inhibits IL-6, TNF-α, and MHC-II production	Promotes M2 polarization	
	SMYD3	Upregulates ALOX15	Promotes M2 polarization	
	PRMT1	Upregulates PPAR γ	Promotes M2 polarization	
HDMs	JMJD3	Upregulates TNF-α and IL-6 after LPS stimulation; upregulates Arg-1, Ym1, Fizz1 and CD206 after IL-4 stimulation	Promotes M1 polarization; promotes M2 polarization	
DNA modifying enzymes				
DNMTs	DNMT1	Activates the JAK2/STAT3 signaling pathway	Promotes M1 polarization	AZA, DEC;
	DNMT3b	Inhibits PPAR γ	Promotes M1 polarization	
TET	TET2	Upregulates inflammatory mediators during the response to LPS	Promotes M1 polarization	DMOG

*HMEs *h**istone modifying enzymes, *HDAC h*istone deacytelase, *HMTs* histone methyltransferases, *HDMs* histone demethylases, *DNMTs* DNA methyltransferases, *TET t*en-eleven translocation enzymes, *SAHA s*uberoylanilide hydroxamic acid, *VPA v*alproic acid, *DZNep* 3-deazaneplanocin, *MCP-1*monocyte chemoattractant protein-1, *MTA m*ethylthioadenosine, *AZA* azacytidine, *DEC d*ecitabine, *DMOG* dimethyloxallyl glycine, *IL* interleukin

### DNA methylation

The nucleosome is the basic unit of chromatin and consists of 147 base pairs of DNA and a histone. On the one hand, chromatin is highly compressed and folded to form chromosomes, which cover naked DNA. On the other hand, DNA packages tightly around histones, which impedes DNA opening by transcription machinery [33]. Therefore, improving chromatin accessibility and loss of the binding between DNA and histones is beneficial for transcription. DNA methylation is an epigenetic mechanism that influences chromatin accessibility and the tightness of the binding between DNA and histones [34]. CpG islands are characterized by CpG-rich regions, and cytosine is always easily methylated to silence gene expression. However, in embryonic stem cells, ~25% of all methylation is in a non-CG context [35]. Non-CG methylation plays an essential role in somatic cell reprogramming, brain development, diabetes and obesity [36]. Promoter and enhancer methylation are generally associated with gene repression that can occur by inhibiting DNA binding with transcription factors or recruiting repressive methyl-binding proteins. In mammals, three types of methyltransferases can add the DNA methylation modification: DNA methyltransferases (DNMT)-1, DNMT-3A and DNMT-3B, while ten eleven translocation (TET) proteins remove the modification [37,38].

**Figure 2:**
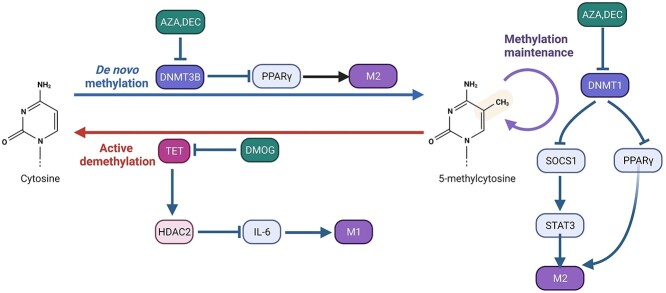
DNA methylation in regulating macrophage polarization. DNA methyltransferase is involved in controlling DNA methylation and downstream transcription factors. The inhibitors that regulate DNA methyltransferase are in green boxes. *AZAa*zacytidine, *DEC* decitabine, *DMOG* dimethyloxallyl glycine, *HDAC2* histone deacetylase2, *TET* ten-eleven translocation enzymes, *DNMT* DNA methyltransferases, *PPAR γ* peroxisome proliferator-activated receptors γ, *SOCS1* socs suppressor of cytokine signaling 1, *STAT3* signal transducers and activators of transcription 3

Both DNMT3b and DNMT1 are associated with M1-like macrophage polarization. DNMT3b targets and inhibits the promoter of PPAR γ1, a positive regulator of M2-like macrophage polarization [39]. The obesity-associated factors saturated fatty acids improve the expression of DNMT3b to enhance the DNA methylation of PPAR γ1 [39]. In addition to inducing methylation, DNMT-3a and DNMT-3b also induce active DNA demethylation under low levels of *S*-adenosyl methionine [37]. Compared with DNMT-3b, DNMT1 preferentially modifies hemimethylated DNA and maintains methylation patterns during replication [40]. DNMT1 mediates promoter hypermethylation of the SOCS1 gene to activate the janus kinase (JAK2)/STAT3 signaling pathway [41]. This is useful for Liver X receptor (LXR), PPAR and STAT3 or LPS to induce the secretion of proinflammatory cytokines, such as TNF-α and IL-6, by increasing the expression of DNMT1 in RAW264.7 cells [41]. Zhang *et al*. [42] reported that loss of TET2 selectively mediates activation of IL-6 transcription in macrophages and that histone deacetylase 2 (HDAC2) is recruited by TET2 to specifically repress histone deacetylation of the IL-6 gene. However, as demethyltransferases, TET family enzymes can oxidize 5-methylcytosine and revert it to cytosine [43]. How TET proteins directly demethylate genes during macrophage polarization is not well understood (Figure 2).

### Histone modifications

The nucleosome, the basic unit of chromatin, consists of histones and DNA. Histones bind with DNA and contain five components: H1, H2A, H2B, H3 and H4 [44]. Modifications of N-terminal tails on histone tails regulate the interaction of histones and DNA to induce or inhibit nucleosome unwinding to form euchromatin or heterochromatin, thereby affecting the affinity of transcription factors and structural gene promoters [45]. Posttranslational modifications, such as methylation, acetylation, phosphorylation and ubiquitination, can be added and removed from histones to regulate transcriptional activity. Histone modifying enzymes (HMEs), which include histone methyltransferases (HMTs), histone demethylases (HDMs), histone acetyltransferases (HATs) and HDACs, write and erase the modifications of histones [46]. The function of histone methylation depends on the methylation site. Lysine methylation of histones can regulate both transcriptional activation and inhibition, while arginine methylation promotes transcriptional activation [47]. Histone acetylation is typically associated with gene activation by weakening histone–DNA interactions to allow access for various transcription factors to specific regions.

Su(var)3–9, enhancer-of-zeste, trithorax7/9 (SET7/9), an HMT member, is a chromatin histone H3-lysine 4 methyltransferase that activates NF-κB to induce the production of TNF-α and monocyte chemoattractant protein 1 (MCP-1) [48]. Another HMT, domain-containing protein (MYND) domain containing 2 (SMYD2), targets the H3 lysine 36 site. In contrast with SET7/9, SMYD2 negatively regulates the production of proinflammatory cytokines, such as IL-6, TNF-α and MHC-II, to inhibit M1 polarization [49]. SMYD3 upregulates ALOX15, a lipoxygenase M2 marker that has been reported by Liu *et al*. [50]. Arginine methyltransferase 1 (PRMT1) plays an essential role in M2 polarization by methylating histone H4R3me2a at the PPAR γ promoter [51]. On the other hand, PRMT1 targets an MHC II-induced protein, Class II transactivator (CIITA), to promote CIITA degradation [52]. Therefore, PRMT1 regulates both M1 and M2 polarization through different mechanisms. Jumonji domain-containing 3 (JMJD3) is an HDM member that acts as a specific demethylase of H3K27 [53]. Several reports have demonstrated that JMJD3^−/−^ macrophages do not show impairment of M1 differentiation, indicating that JMJD3 may not be involved in M1-like macrophage programming [53,54]. Another study reported that JMJD3 participates in the transcriptional output at low intensity and is independent of H3K27me3 demethylation. This suggests that JMJD3 makes fine adjustments to the transcription rates rather than being indispensable for them [55]. Although JMJD3 is dispensable for M1 polarization, it is important for M2 polarization. JMJD3 contributes to maintenance of M2 marker genes, such as Arginase-1 (Arg-1), Chi3l3 and Retnla, in a transcriptionally active state and removes the repressive H3K27me marks on IRF4, a regulatory protein in M2 polarization [54,56].

P300/CBP is the most studied HAT targeting H3K9 [57]. Li *et al*. [58] reported that P300 acetylating X-box protein 1 induces the activation of homocysteine-inducible endoplasmic reticulum protein with ubiquitin-like domain 1, a transmembrane protein skewing M2 polarization in RAW264.7 cells. For M1 polarization, P300/CBP enhances the transcription levels of Kruppel-like factor 2 (KLF2) and KLF4. Both inhibit M1 polarization by weakening the NF-κB signaling pathway [59]. HDACs include four distinct classes: Classes I, II, III and IV. HDAC3, a member of Class I, supports M1-like macrophage activation, which is essential for the production of hundreds of inflammatory cytokines, such as IL-6 and IFN-β, by influencing STAT1 [60]. Loss of HDAC3 promotes macrophage skewing toward the M2 phenotype after stimulation by Th2 cytokines [61]. Similar to HDAC3, HDAC9 also inhibits M2 polarization by deacetylating the PPAR γ promoter [62,63]. Silent information regulator 2 homolog (SIRT1) is a negative regulator of M1 polarization via inhibition of the NF-κB pathway [64]. HDAC4 deacetylates histone 3 on STAT6 proteins to activate Arg-1 transcription when cells are stimulated with IL-4. Under LPS and IFN-γ stimulation, HDAC4 inhibits NF-κB [38]. Bromodomain extra terminal (BET) proteins include Brd2, Brd3 and Brd4, which are responsible for reading histone acetylation marks to recruit TFs for gene transcription [65]. Belkina *et al*. [66] reported that BET proteins play an important role in proinflammatory cytokine production in macrophages.

**Figure 3:**
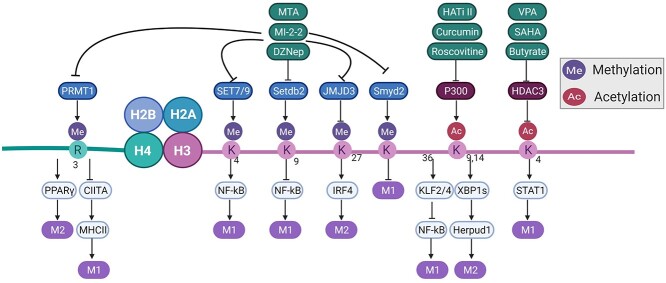
A portion of the histone modifications that participate in regulation of macrophage polarization. Histone modification enzymes involved in controlling macrophage polarization and downstream transcription factors. The modification site and modification type are shown on H3 and H4. The inhibitors that regulate histone modification are presented in green boxes. *MTAm*ethylthioadenosine, *MI-2-2* MLL–menin interaction inhibitor-2-2, *DZNep* 3-deazaneplanocin3, *VPA* valproic acid, *SAHA* suberoylanilide hydroxamic acid, *HATi II* histone acetyltransferase inhibitor II, *SET7/9* Su(var)3–9, enhancer-of-zeste, trithorax7/9, *SMYD* SET and MYND domain containing, *SETDB2* SET domain bifurcated histone lysine methyltransferase 2, *JMJD3* Jumonji domain-containing 3, *HDAC* histone deacytelase, *PRMT1 p*rotein arginine methyltransferase 1, *CIITAc*lass II transactivator, *PPAR γ* peroxisome proliferator-activated receptors γ, *IRF4* IFN-regulatory factor 4, *STATs s*ignal transducers and activators of transcriptions, *KLF2* Kruppel-like factors 2, *XBP1s* X-box protein 1, *Herpud1* homocysteine inducible endoplasmic reticulum protein with ubiquitin-like domain 1

DNA methylation and histone modifications always cooperate to form a regulatory network. For example, the DNMT inhibitor 5-aza-2-deoxycytidine (AZA) and the HDAC inhibitor trichostatin A (TSA) can activate STAT3, which inhibits the expression of JMJD3 [67,68]. JMJD3 is directly regulated by NF-κB, and SET7/9 activate NF-κB. Both of them form a mutually influencing mechanism [48] (Figure 3).

### ncRNA

ncRNA is different from mRNA because it does not encode a protein, the traditional way to express genetic information [69]. However, ncRNAs regulate gene expression on other levels, e.g. by enhancing or blocking transcription or translation, altering the splicing of mRNA, or recruiting HMEs, such as polycomb group complex members. Among ncRNAs, microRNAs (miRNAs) are the most comprehensive [70]. miRNAs bind target mRNA and silence it, forming a complex network with TFs to organize gene expression and maintain the balance between M1 and M2 macrophages. Under the stimulation of IL-13 or TGF-β, miR-155 targets IL-13Rα1 and SMAD2 separately to skew M1-like genes [71]. However, after stimulation with IFN-γ, miR-155 suppresses NO production by targeting CCAAT/enhancer binding protein β [72]. miRNAs are also capable of indirectly modulating gene expression by acting on other epigenetic modulators. For example, miR-145 promotes the production of IL-10 by targeting HDAC11, a gene silencer of IL-10 [73]. In brief, many miRNAs and their synthetic substitutes have been demonstrated to promote wound healing [74,75].

Other than miRNAs, most research focuses on long noncoding RNAs (lncRNAs) [76]. LPS-induced M1 macrophages express more lncRNA cyclooxygenase-2 (cox-2) than IL-4-induced M2 macrophages, and suppression of cox-2 decreases the level of M1 macrophage markers but increases that of M2 macrophage markers [77]. Another lncRNA, Mirt2, is also induced by LPS. Mirt2 decreases Lys63 (K63)-linked ubiquitination of tumor necrosis factor receptor-associated factor6 (TRAF6), a ubiquitin ligase that is a key mediator of LPS-induced inflammation. Some lncRNAs regulate macrophage polarization by interacting with miRNAs [78]. For example, lncRNA MEG3 prevents M2 macrophage polarization via the miR-223/TRAF6/NF-κB axis [79]. The lncRNA NEAT1 induces macrophage M2a polarization via the miR-224-5p/IL-33 axis [80]. After burn injury, lncRNA XIST inhibits miR-19b to promote M2 polarization to accelerate wound healing [81].

Another type of ncRNA, circular RNAs (circRNAs), have a covalently closed loop structure. Recently, many studies have focused on the relationship between circRNAs and macrophages. circRNA Cdyl promotes M1 polarization by inhibiting nuclear translocation of IRF4 [82]. After stimulation with LPS, circRNA PPM1F enhances the NF-κB signaling pathway to promote M1 polarization [83]. Some circRNAs act as molecular sponges for miRNAs. hsa_circ_0005567 induces M2 polarization via the miR-492/SOCS2 axis [84], and circRNA HIPK3 induces inflammatory cytokines in macrophages by sponging miR-192 and miR-561 [85]. In wound healing, circRNA nhg11 promotes M2 macrophage polarization via the miR-144-3p/hypoxia-inducible factor (HIF)-1α axis [86].

The relationship between macrophages and other ncRNAs, such as Smallinterfering RNA (siRNA) and Piwi-interacting RNA (piRNA), has rarely been reported. The function of ncRNA in wound healing deserves further study.

### Epigenetic regulation of macrophage phenotype by polarizing stimuli in normal wound healing

Normal wound healing proceeds through four discrete phases: hemostasis, inflammation, proliferation and remodeling. In the early inflammatory phase, monocytes are recruited to the wound site by chemokines. The recruited monocytes then differentiate into M1 macrophages in response to pathogen-associated molecular patterns or damage-associated molecular patterns to trigger a potent immune response and thoroughly clear pathogens and damaged tissues. After eliminating pathogens, necrotic tissues and neutrophil corpses via phagocytosis, M1 macrophages transition into the M2 phenotype to promote wound healing. Polarization of macrophages into the M1 or M2 phenotype involves a complex network of epigenetic regulation induced by various stimuli. Different stimuli lead to different epigenetic mechanisms and macrophage activation outcomes [7,87]. Despite the fact that there is currently limited information on the detailed epigenetic regulation mechanism triggered by each stimulus, we attempted to summarize some widely acknowledged stimuli that can cause epigenetic alterations in macrophages during the wound healing process.

For stimuli that promote M1 macrophage activation via epigenetic regulation, Toll-like receptor ligands and Th1 cytokines, such as LPS and IFN-γ, individually or in combination, can induce M1 macrophage activation by influencing the epigenetic process. In murine bone marrow-derived macrophages (BMDMs), LPS can influence the mRNA levels of some members of the HDAC family. LPS can transiently inhibit and then induce the expression of many HDACs (HDACs 1, 4, 5, 7, 8), which leads to the upregulation of proinflammatory genes [88]. LPS-treated macrophages can also recruit JMJD3, an H3K27me3 demethylase in the jumonji family, to the TNF-α and IL-6 promoters to upregulate their expression [55]. SMYD2, a HMT that suppresses IL-6, TNF-α and MHC-II expression, is also downregulated in response to LPS stimulation [49]. The histone mark H3K4me3, which can be enriched in the M1 marker gene CXCL10 promoter region by HMT in myeloid lymphoid leukemia (MLL), is significantly upregulated in LPS- and IFN-γ-treated M1 macrophages [89]. TNF-α is another polarizing factor that can cause epigenetic alterations in macrophages. The histone acetyltransferase ‘males absent on the first’ (MOF) in macrophages, which targets H4K16 to induce a proinflammatory response, is significantly upregulated following TNF-α stimulation. The upregulated MOF promotes the transcription of NF-κB–mediated inflammatory genes in macrophages [90]. TNF-α also upregulates SET7/9, a histone H3K4 methyltransferase, to promote the expression of NF-κB-dependent inflammatory genes in macrophages [48].

To limit inflammation and favor progression of the healing process, macrophages must switch from the proinflammatory M1 phenotype to the anti-inflammatory M2 phenotype. Th2 cytokines, such as IL-4 and IL-13, are generally considered to regulate this phenotypic switch [91,92]. Studies indicate that IL-4 and IL-13 promote phenotypic switching through epigenetic modifications. Mullican *et al*. [61] reported that inflammatory genes that are upregulated in IL-4-treated wild-type macrophages also exhibit increased expression in unstimulated macrophages with HDAC3 deletion. These results suggest that HDAC3 restrains the activation of M2 macrophages by inhibiting the expression of a subset of genes upregulated by IL-4 while favoring activation of the M1 phenotype. Chromatin remodeling also plays an important role in induction of the M2 phenotype by IL-4. Ishii *et al*. [56] reported that IL-4 stimulation contributes to higher expression of STAT6-mediated JMJD3 in macrophages. An increase in the JMJD3 level is conducive to reduced H3K27me2/3 deposition and promotes the transcriptional upregulation of specific M2 genes. SMYD3, an H3K4 methyltransferase, has been demonstrated to promote M2 macrophage polarization. The level of SMYD3 is upregulated in human monocyte-derived macrophages exposed to M-CSF, IL-4 and IL-13 but downregulated in macrophages exposed to M1 stimulation factors. The lipoxygenase M2 marker ALOX-15 shows significantly increased methylation levels and transcriptional activation after upregulation of SMYD3 [89].

### Epigenetic regulation of macrophages by detrimental stimuli in chronic wound healing

Chronic wounds are defined as wounds that do not heal for >3 months [10]. Generally, chronic wounds can be classified into diabetic foot ulcers, pressure ulcers and vascular ulcers [93]. Different from the well-orchestrated normal wound healing process, chronic wounds fail to proceed through the inflammatory phase to the proliferation phase [94]. The pathophysiology of chronic wounds is complicated, and various factors, such as hyperglycemia status, venous insufficiency, arterial hyperperfusion and persistent pressure, are involved [95]. What these chronic wounds have in common includes persistent inflammation, repeated infection or formation of biofilms, stalled re-epithelialization, impaired angiogenesis, accumulation of excessive senescent cells and overproduction of reactive oxygen species (ROS) [96–98]. The hallmark of most chronic wounds is the existence of chronic and persistent inflammation. Compared with normal wounds, in the early inflammatory phase, M1 macrophages in chronic wounds fail to effectively clear necrotic tissues and pathogens due to decreased bactericidal and phagocytic activities caused by detrimental stimuli in chronic wounds [99]. Macrophage dysfunction and the prolonged presence of proinflammatory stimuli (necrotic tissues, pathogens, neutrophil corpses, senescent cells) amplify local inflammation, causing persistent inflammation and impeding macrophage transition from the M1 phenotype to the M2 phenotype [100]. As a result, wounds suffer from elevated levels of proinflammatory cytokines and matrix metalloproteinases. Macrophage dysfunction could be the consequence of a mutual reaction between inherent genetic changes and epigenetic alterations caused by environmental stimuli. Treatment targeting detrimental environmental stimuli that cause epigenetic alterations in macrophages in chronic wounds is more practical and feasible than treatment targeting inherent genetic changes. To improve chronic wound healing, it is of great significance to determine the exact detrimental stimuli and underlying epigenetic mechanisms that cause macrophage dysfunction in the early stage of wounds. Herein, we attempt to summarize some detrimental stimuli that can exert epigenetic regulation in macrophages in chronic wounds.

In diabetic wounds, transient hyperglycemia can promote H3K4 methylation in the proximal promoter region of NF-κB via the methyltransferase SETD7. As a consequence, the expression levels of monocyte MCP-1 and vascular adhesion molecule-1 are increased [101]. Brasacchio *et al*. [102] demonstrated that hyperglycemia can mediate decreased dimethylation and trimethylation of H3K9 and increased monomethylation of H3K4, thus causing increased NF-κB gene expression. Additionally, hyperglycemia can decrease trimethylation of H3K9 at the promoter region of IL-6 in human monocytes, resulting in increased IL-6 expression [103]. Furthermore, hyperglycemia can affect the expression of certain miRNAs. For example, it has been reported that miR-146a is downregulated in peripheral blood mononuclear cells isolated from diabetic patients [104]. The decreased level of miR-146a in diabetic wound macrophages fails to downregulate the expression of inflammatory genes, causing a prolonged inflammatory reaction [105]. Kimball *et al*. compared macrophages isolated from wounds of diabetic patients and healthy people and found decreased expression of the methyltransferase SET domain bifurcated histone lysine methyltransferase 2 (SETDB2) in diabetic wounds. SETDB2 specifically trimethylates H3K9me3 at the NF-κB binding site, making the binding site inaccessible to transcription factors and thereby inhibiting proinflammatory gene expression. SETDB2 expression in wound macrophages is regulated by IFN-β. In diabetic wounds, the impaired IFN-β–SETDB2 axis results in persistent pro-inflammatory macrophage phenotypes [106]. Davis *et al*. [107] reported increased MLL1-mediated H3K4 trimethylation of the CytosolicPhospholipaseA_2_ (cPLA_2_) promoter to upregulate cPLA_2_ gene expression and increased TGF-β1-induced miR-29b-mediated hypomethylation of the Cox-2 gene promoter via destabilization of DNMT3a/b to upregulate COX-2 levels. The elevated COX-2/PGE_2_ levels result in upregulation of downstream macrophage-mediated inflammation in diabetic wounds.

Chronic wounds generally suffer from long-term hypoxia due to blood circulation disorder, vascular pathological changes and impaired angiogenesis. Hypoxia can cause epigenetic changes in macrophages. HIF is the key mediator of the hypoxia response in macrophages. The expression of inflammatory cytokines (TNF-α, IL-6, IL-1β) in macrophages is induced by hypoxia in a HIF-1α-dependent and HIF-1α-independent manner. Critically, HIF-1α can interact with HAT P300/CBP to modulate the histone acetylation of HIF-1α target genes [108]. Furthermore, hypoxia limits the activity of the jumonji family HDMs, inducing an increase in H3K9me2 and H3K9me3 at the promoter region of chemokine receptor (CCR1), CCR5 and CCL2, which is in accordance with the reduced expression of the corresponding mRNA under hypoxia [109]. Paradoxically, hypoxia can induce inflammatory macrophages, while it also appears to inhibit the recruitment of inflammatory monocytes via downregulation of CCL2 expression.

### Treatments targeting detrimental stimuli in the microenvironment to improve wound healing

Considering the adverse effects of detrimental stimuli on wound healing, treatments targeting detrimental stimuli in the wound microenvironment are of great significance for wound healing. For normal wound healing, it is necessary to avoid the existence of detrimental stimuli in the wounds. Wound treatments that include antibacterial and antioxidative effects and relief of hypoxia/ischemia are beneficial for normal wound healing [110]. For chronic wounds, due to the complex pathophysiology caused by various factors, such as hyperglycemia status, venous insufficiency, arterial hyperperfusion and persistent pressure, it is crucial to target these causes by controlling blood glucose, improving vascular blood circulation or removing persistent pressure [111]. In addition, due to the prolonged existence of proinflammatory stimuli (necrotic tissues, biofilm, neutrophil corpses, senescent cells) caused by the reduced bactericidal and phagocytic capacity of macrophages in chronic wounds, clinical wound debridement treatment can establish a ‘fresh new’ wound and reactivate the re-epithelialization process [10,112].

### Pharmacological modulators targeting epigenetic enzymes to influence macrophage phenotype

Given the crucial role of epigenetics in regulating macrophage phenotypes, there exists great potential to target specific epigenetic enzymes for therapeutic intervention to regulate macrophage phenotypes and promote wound healing. There are various pharmacologic modulators for different types of epigenetic enzymes, some of which target specific enzymes, while others target a wide range of enzymes. A number of diverse pharmacologic modulators are commercially available to modulate the epigenetic activity of macrophages, many of which are clinically utilized to treat cancer or other diseases. Despite the fact that there are currently limited studies on the effect of epigenetic enzyme inhibitors on wound healing, it is important to summarize the potential pharmacologic modulators that might be used to target specific epigenetic enzymes to regulate macrophage phenotypes in the near future (Table 2).

**Table 2:** Pharmacologic modulators targeting epigenetic enzymes to influence macrophage phenotype

**Category**	**Pharmacologic modulator**	**Effect on macrophages**
DNMTis	AZA	Reduces the expression of iNOS and NO; increases the expression of Arg-1 and Fizzl
	DEC	Reduces the expression of TNF-α, IL-1α, IL-1β, IL-6, iNOS, CCL2, CCL5 and CCL9; increases the expression of Arg-1, CD206, Nos2, STAT3 and SOCS1
TETis	DMOG	Reduces the expression of iNOS and NF-κB activation; increases the expression of Fizz1, Arg-1 and Ym1
HMTis	DZNep	Reduces the expression of TNF-α
	MI-2-2	Reduces the expression of CXCL10
	MTA	Reduces the expression of TNF-α and IL-6
HATis	HATi II	Reduces the expression of IL-1β
	Roscovitine	Reduces the expression of iNOS, COX-2, and NO and NF-κB activation
	Curcumin	Reduces the expression of COX-2, CCL2, TNF-α, and IL-6 and NF-κB activation; increases the expression of PPAR γ, CD36, CD206 and Arg-1
HDACis	Vorinostat	Inhibits the polarization of macrophages stimulated with LPS and IFN-γ
	VPA	Reduces the M1 phenotype *in vitro*
	Butyrate	Increases the phosphorylation of STAT6 and the expression of Fizz1, Ym1, Arg-1 and CD206; reduces the expression of TNF-α, IL-6 and MCP-1

*AZA* *a*zacytidine, *DEC d*ecitabine, *DMOG* dimethyloxallyl glycine, *DZNep* 3-deazaneplanocin, *MTA* methylthioadenosine, *HATi II* histone acetyltransferase inhibitor II, *VPA* valproic acid, *HMEs H*istone modifying enzymes, *HDAC h*istone deacytelase, *HMTs* histone methyltransferases, *HDMs* histone demethylases, *DNMTs* DNA methyltransferases, *TET t*en-eleven translocation enzymes

### DNMT inhibitors

AZA and decitabine (DEC) are two well-recognized DNMT inhibitors that are used in the clinical treatment of myelodysplastic syndrome [113–115]. Many studies have demonstrated the effectiveness of AZA and DEC in promoting M1 macrophage polarization to the M2 phenotype.

In M1 macrophages, AZA can reduce the expression of M1 markers and increase the expression of M2 markers. It has been reported that AZA treatment inhibits the expression of iNOS and NO in LPS-treated RAW264.7 cells [116], peptidoglycan (PGN)-treated RAW264.7 cells [117] and LPS- and IFN-γ-treated murine peritoneal macrophages *in vitro* [118]. AZA at a concentration of 10 μM significantly inhibited the expression of iNOS in PGN-stimulated RAW264.7 cells after 24 h of treatment *in vitro*. Furthermore, the expression levels of the M2 markers Arg-1 and Fizz1 were found to be increased in PGN-treated RAW264.7 cells [117]. Intraperitoneal administration of AZA significantly ameliorated cardiac injury in a mouse myocardial infarction model by promoting macrophages toward the M2 phenotype via iNOS inhibition [117]. Another study reported that the cardioprotective effect of AZA in myocardial infarction was associated with modulation of sumoylation of IRF1 to inhibit iNOS expression in macrophages *in vivo* [116]. It has also been reported that AZA can promote cutaneous wound healing by increasing cell proliferation, collagen deposition and stem cell recruitment [119]. Topical administration of AZA at a 10 mM concentration once per day significantly accelerated wound healing in a rat full-thickness wound model by promoting wound reepithelization and cell proliferation via increased TGF-β expression and decreased TNF-α and IL-6 expression [120].

DEC has effects similar to those of AZA in unstimulated and M1 macrophages. DEC reduces the expression of the M1 markers TNF-α, IL-1β, IL-6 and iNOS and many other chemokines (including CCL2, CCR2, CCL5 and CCL9) in various LPS-treated macrophages and in macrophages isolated from atherosclerotic plaques [121,122].

Pretreatment of RAW264.7 cells with 0.5 μM DEC inhibited the expression of proinflammatory cytokines after LPS stimulation *in vitro*. Low-dose injection of DEC ameliorated the development of atherosclerosis via demethylation of the LXRα and PPAR γ1 promoters to suppress macrophage inflammation *in vivo* [121]. The expression of nitric oxide synthase (Nos2) was decreased and the expression of CD206 was increased in LPS-stimulated BMDMs treated with DEC *in vitro*. The number of M1 macrophages was decreased and the number of M2 macrophages was increased in the lung tissues of LPS-treated acute lung injury mice post-treatment with DEC [122]. This study also demonstrated that the effect of DEC on the expression of CD206, Nos2 and Arg-1 was more prominent when DEC was combined with TSA [122]. In addition, the combined use of TSA and DEC increased the phosphorylation of STAT3 in LPS-treated BMDMs, thereby downregulating M1 inflammatory levels [122]. Additionally, it was reported that the LXRα, PPAR γ and STAT3 pathways can decrease the expression of M1 markers, such as CCL2, CCL5, TNF-α and IL-6 [62]. In LPS-treated RAW264.7 cells, DEC promoted the expression of SOCS1, which positively regulates the LXRα, PPAR γ and STAT3 pathways [41], thereby downregulating M1 inflammation levels.

### TET protein inhibitors

Dimethyloxallyl glycine (DMOG), a TET protein inhibitor, has been reported to attenuate LPS-induced endotoxic shock and promote M2 macrophage polarization *in vivo* [123]. Peritoneal macrophages isolated from mice intraperitoneally injected with DMOG (8 mg/mouse) before LPS treatment showed significantly lower NF-κB activity and iNOS expression. Compared with mice treated with LPS alone, the serum levels of TNF-α and IL-10 were significantly decreased and increased, respectively, in mice pretreated with DMOG before LPS treatment. Furthermore, DMOG facilitated M2 polarization in murine peritoneal macrophages collected from mice exposed to chitin or LPS *in vivo*. In addition, 1 mM DMOG increased the expression of Arg-1, Relm-α and Ym1 in *in vitro* cultured peritoneal macrophages stimulated with LPS and IFN-γ, IL-4 and IL-13, or IL-10 [123]. Notably, DMOG conversely promoted NF-κB activity and iNOS expression in unstimulated murine peritoneal macrophages [123]. These results have profound implications for the effect of TET proteins on regulating M2 macrophage polarization.

### HMT inhibitors

The effects of relatively few HMT inhibitors on the regulation of macrophages have been reported. 3-Deazaneplanocin (DZNep) is an enhancer of zeste homolog 2 (EZH2) inhibitor that has been reported to inhibit the production of TNF-α in LPS-treated RAW264.7 cells *in vitro*. It has been reported that DZNep exhibits no cytotoxicity at concentrations between 1 and 100 μM. In addition, 100 μM DZNep inhibited the production of TNF-α in LPS-treated RAW264.7 cells by 86% compared with the control group [124]. MLL–menin interaction inhibitor-2-2 (MI-2-2) has been reported to reduce the expression of CXCL10 in IFN-γ-stimulated human monocyte-derived macrophages *in vitro*. Macrophages pretreated with 40 μM MI-2-2 24 h prior to IFN-γ stimulation showed a decreased number of CXCL10-positive cells [89]. Methylthioadenosine (MTA), an HMT inhibitor, inhibited the TNF-α mRNA level and the secretion of TNF-α and IL-6 in LPS- and INF-γ-treated BMDMs at a 0.5 mM concentration *in vitro*. Paradoxically, MTA upregulated the mRNA level of IL-1β but had no impact on the mRNA levels of Nos2, IL-6 and STAT1 [125]. Other studies testing MTA against M1 macrophages also found that 0.5 mM MTA can inhibit the expression of iNOS and TNF-α in LPS-stimulated RAW264.7 cells and BMDMs *in vitro* [126,127].

### HAT inhibitors

Some HAT inhibitors (HATis) have been reported to regulate the M1 phenotype, and different effects have been reported. HATi II downregulates the M1 phenotype by reducing IL-1β secretion [128]. Roscovitine at a concentration of 25 μM inhibited LPS-induced expression of iNOS, COX-2, IL-6 and IL-1β by inhibiting NF-κB activation in RAW264.7 cells *in vitro* [129]. Curcumin, a P300 inhibitor, has been reported to reduce the M1 phenotype by inhibiting the activity of NF-κB, the production of ROS and the expression of COX-2, CCL2, TNF-α and IL-6 [130–133]. Simultaneously, 12.5 μM curcumin increased PPAR γ and CD36 expression in RAW264.7 cells treated with LPS and IFN-γ *in vitro* [134]. Studies have also found that curcumin can promote the M2 phenotype in unstimulated macrophages. Unstimulated RAW264.7 cells showed increased levels of IL-4, IL-13, PPAR γ, CD206 and Arg-1 after curcumin treatment [135]. Curcumin is one of the few epigenetic enzyme inhibitors that have been widely reported in chronic wound healing. Due to the antioxidant and anti-inflammatory activity of curcumin, it has been commonly incorporated into wound dressings to regulate inflammation and promote wound healing [136,137]. Recently, it has been reported that a curcumin-incorporated 3D bioprinting gelatin methacryloyl hydrogel reduced ROS-induced adipose-derived stem cell apoptosis and improved implantation survival in diabetic wounds [138].

### HDAC inhibitors

HDAC inhibitors (HDACis) are the most widely detected epigenetic modulators in regulating macrophage phenotypes. It has been reported that topical administration of TSA on wounds can specifically accelerate wound healing and enhance monocyte and macrophage populations in the wound bed *in vivo* [139]. Vorinostat, a prominent pan-HDACi, has been demonstrated to significantly inhibit the polarization of macrophages stimulated with LPS and IFN-γ [140,141]. Vorinostat inhibited the release of some inflammatory mediators (IL-12p40 and IL-6) in macrophages at low concentrations (<3 μM) but promoted the production of other cytokines at higher concentrations (>3 μM) *in vitro*. Similar results were demonstrated in a rat arthritis model *in vivo*, where vorinostat exhibited a therapeutic effect only at a low dose [141]. Valproic acid (VPA), another pan-HDACi, has been reported to prominently reduce the M1 phenotype *in vitro* [142,143]. VPA at a 2 mM concentration inhibited the phosphorylation of PI3K/Akt/murine double minute2 (MDM2) signaling in RAW264.7 cells, thus inhibiting NF-κB transcriptional activation in response to LPS [143]. Butyrate, a pan-HDACi that has been used in clinical trials for schizophrenia, increased the phosphorylation of STAT6 and the expression of Fizz1, Ym1, Arg-1 and CD206 in IL-4-stimulated murine BMDMs *in vitro* [144]. Furthermore, oral administration of butyrate reduced the adhesion and migration of macrophages, thus inhibiting the progression of atherosclerosis [145]. *In vitro* experiments demonstrated that butyrate can reduce the expression of proinflammatory mediators in LPS-stimulated M1 macrophages [146,147]. Additionally, butyrate also reduced the expression of TNF-α, IL-6 and MCP-1 in RAW264.7 cells cocultured with 3 T3-L1 adipocytes by inhibiting the phosphorylation of mitogen-activated protein kinasesmitogen-activated protein kinases (MAPK) and IκB-α [148].

## Conclusions

Macrophages are the major immune cells in wound healing and have high plasticity. Understanding the factors that regulate their function is of critical importance. However, investigation of the epigenetic regulation of macrophage plasticity and wound healing is still at an early stage. Exploring the differential expression of epigenetic enzymes, especially HMEs and ncRNAs, between macrophages in normal and chronic wounds is essential. Since epigenetics plays an essential role in macrophage plasticity, environmental stimuli and pharmacologic modulators targeting epigenetic enzymes could be potential therapeutic targets for wound healing. However, the function of certain environmental factors in wounds, such as mechanical force and tissue debris, in wound cellular epigenetic regulation is still unclear. Individual epigenetic enzymes have differential effects on different tissue macrophage inflammatory responses. Inhibitors with high specificity should be screened. For mechanistic research, the specific modification sites of epigenetic enzymes and TFs should be identified. Wound healing is a dynamic process and macrophages are changing constantly, but how epigenetics affects the dynamics of macrophages in normal wounds is unclear.

## Abbreviations

AZA: Azacytidine; BET: Bromodomain extra terminal; CIITA: Class II transactivator; CircRNAs; Circular RNAs; DEC: Decitabine; DMOG: Dimethyloxallyl glycine; DNMT: DNA methyltransferases; DZNep: 3-Deazaneplanocin; HAT: histone acetyltransferase; HATi II: Histone acetyltransferase inhibitor II; HDAC: Histone deacytelase; HDACis: Histone deacetylase inhibitors; HDM: Histone demethylase; HIF: Hypoxia-inducible factor; HMEs: Histone modifying enzymes; HMTis: Histone methyltransferase inhibitors; IRF: IFN regulatory factor; JMJD3: Jumonji domain-containing 3; KLF2: Kruppel-like factor 2; lncRNAs: Long noncoding RNAs; MCP-1: Monocyte chemoattractant protein 1; MiRNAs: MicroRNAs; MLL: Myeloid lymphoid leukemia; MI-2-2: MLL–Menin interaction inhibitor-2-2; MOF: Males absent on the first; MTA: Methylthioadenosine; ncRNA: Noncoding RNA; PPAR γ: Peroxisome proliferator-activated receptors γ; PRMT1: Protein arginine methyltransferase 1; PTMs: Posttranslational modifications; ROS: Reactive oxygen species; SAHA: Suberoylanilide hydroxamic acid; SET7/9: Su(var)3–9, enhancer-of-zeste, trithorax7/9; SETDB2: SET domain bifurcated histone lysine methyltransferase 2; SIRT1: Silent information regulator 2 homolog; SMYD: SET And MYND domain containing; SOCS: Suppressors of cytokine signaling; STATs: Signal transducers and activators of transcriptions; TET: Ten-eleven translocation enzymes; TFs: Transcription factors; TNF-α: Tumor necrosis factor alpha; TRAF6: Tumor necrosis factor receptor-associated factor6; TSA: trichostatin A; VPA: Valproic acid.
